# Clinical features of adolescent-onset functional motor disorders in tertiary movement disorders centers

**DOI:** 10.1007/s00415-026-13761-w

**Published:** 2026-03-22

**Authors:** Christian Geroin, Tommaso Ercoli, Enrico Marcuzzo, Angela Sandri, Serena Camozzi, Domenico Maddaloni, Luigi Michele Romito, Roberto Eleopra, Lucia Tesolin, Serena Pellegrin, Alessandra Nicoletti, Giovanni Mostile, Alessandro Magliozzi, Andrea Pilotto, Nicola Modugno, Enrica Olivola, Benedetta Demartini, Veronica Nisticò, Roberto Erro, Sofia Cuoco, Alessandro Tessitore, Rosa De Micco, Roberto Ceravolo, Eleonora Del Prete, Carlo Dallocchio, Carla Arbasino, Marcello Esposito, Trinchillo Assunta, Francesco Bono, Angelo Pascarella, Martina Petracca, Paola Zinzi, Giovanni Fabbrini, Gina Ferrazzano, Laura Bonanni, Sara Varanese, Alberto Albanese, Paola Polverino, Valentina Baglioni, Giovanni Defazio, Maurizio Zibetti, Paolo Manganotti, Paolo Solla, Giovanna Calandra-Buonaura, Antonio Pisani, Michele Tinazzi

**Affiliations:** 1 Department of Surgery, Dentistry, Paediatrics and Gynaecology, University of Verona, P.le Scuro 10, 37134 Verona, Italy; 2 Neurology Unit, Movement Disorders Division, Department of Neurosciences, Biomedicine and Movement Sciences, University of Verona, P.le Scuro 10, 37134 Verona, Italy; 3 Neurology Unit, AOU Sassari, University of Sassari, Sassari, Italy; 4 Parkinson and Movement Disorders Unit, Department of Clinical Neurosciences, Fondazione IRCCS Istituto Neurologico Carlo Besta, Milan, Italy; 5 FND Outpatients Clinic, Neurology and Stroke Unit, General Hospital of Bolzano, Bolzano, Italy; 6 Child Neurology and Neurorehabilitation Unit, Department of Pediatrics, Provincial Hospital of Bolzano (SABES-ASDAA), Lehrkrankenhaus der Paracelsus Medizinischen Privatuniversität, Bolzano-Bozen, Italy; 7 Department G.F. Ingrassia, Section of Neurosciences, University of Catania, Catania, Italy; 8 Oasi Research Institute - IRCCS, Troina, Italy; 9 Neurology Unit, Department of Clinical and Experimental Sciences, University of Brescia, Brescia, Italy; 10 Laboratory of Digital Neurology and Biosensors, University of Brescia, Brescia, Italy; 11 Neurology Unit, Department of Continuity of Care and Frailty, ASST Spedali Civili Brescia Hospital, Brescia, Italy; 12 Division of Clinical Geriatrics, Department of Neurobiology, Care Sciences and Society (NVS), Center for Alzheimer Research, Karolinska Institutet, Solna, Sweden; 13 Brain Health Center, University of Brescia, Brescia, Italy; 14 IRCCS Neuromed, Pozzilli, Italy; 15 Aldo Ravelli Research Center for Neurotechnology and Experimental Brain Therapeutics, University of Milan, Milan, Italy; 16 SC Psichiatria, ASST Santi Paolo e Carlo, Presidio San Paolo, Milan, Italy; 17 Department of Health Sciences, University of Milan, Milan, Italy; 18 Department of Medicine, Surgery and Dentistry Scuola Medica Salernitana, Center for Neurodegenerative Diseases (CEMAND), University of Salerno, Baronissi, Salerno Italy; 19 Department of Advanced Medical and Surgical Sciences, University of Campania “Luigi Vanvitelli”, Naples, Italy; 20 Centro Clinico Malattie NeuroDegenerative, Azienda Ospedaliero Universitaria Pisana, Pisa, Italy; 21 S.C. Neurologia, Dipartimento di Area Medica Specialistica, ASST Pavia, Pavia, Italia; 22 Clinical Neurophysiology Unit, Cardarelli Hospital, Naples, Italy; 23 Department of Medical, Motor and Wellness Sciences, University “Parthenope”, Naples, Italy; 24 Neurology Unit, Botulinum Toxin Therapy Center, Academic Hospital, A.O.U. “R. Dulbecco”, Catanzaro, Italy; 25 Department of Medical and Surgical Sciences, Magna Graecia University, Catanzaro, Italy; 26 UOC Neurologia, Fondazione Policlinico Universitario Agostino Gemelli IRCCS, Rome, Italy; 27 Clinical Psychology Unit, Fondazione Policlinico Universitario Agostino Gemelli IRCCS, Rome, Italy; 28 Department Human Neurosciences, Sapienza University of Rome, Rome, Italy; 29 Department of Medicine and Aging Sciences, University G. d’Annunzio of Chieti-Pescara, Pescara, Italy; 30 Clinica Neurologica Ospedale di Vasto, Chieti, Italy; 31 Department of Neurology, IRCCS Fondazione Istituto Neurologico C. Mondino, Pavia, Italy; 32 Department of Neuroscience, Università Cattolica del Sacro Cuore, Milan, Italy; 33 Department of Neurology, IRCCS Humanitas Research Hospital, Rozzano, Italy; 34 Unit of Child Neurology and Psychiatry, Department of Human Neuroscience, Sapienza University of Rome, Rome, Italy; 35 Department of Translational Biomedicine and Neuroscience, Aldo Moro University of Bari, Bari, Italy; 36 Department of Neurosciences Rita Levi Montalcini, University of Turin, Turin, Italy; 37 SC Neurologia 2U, AOU Città Della Salute e Della Scienza, Turin, Italy; 38 Department of Medical Surgical and Health Sciences Cattinara Hospital, University of Trieste, Trieste, Italy; 39 IRCCS Istituto Delle Scienze Neurologiche di Bologna, Bologna, Italy; 40 University of Bologna, Bologna, Italy; 41 Department Brain and Behavioral Sciences, University of Pavia, Pavia, Italy; 42 IRCCS Mondino Foundation, Pavia, Italy; 43 IRCCS Synlab SDN, Naples, Italy

**Keywords:** Functional neurological disorders, Functional motor disorders, Diagnosis, Adolescents

## Abstract

**Background:**

Functional Motor Disorders (FMDs) represent a diagnostic and therapeutic challenge in pediatric neurology, particularly among adolescents. Their clinical presentation is common but often nonspecific, leading to frequent misdiagnoses and diagnostic delays. We aimed to characterize FMDs in adolescents and to examine the frequency of isolated and combined phenotypes and their associations with demographic and clinical variables.

**Methods:**

In this observational study, data were obtained from the Italian Registry of FMDs, including patients with a clinically definite diagnosis of FMD consecutively enrolled at 25 Italian tertiary movement disorders centers.

**Results:**

Among 847 patients, 93 (10.9%) had adolescent-onset FMDs. Motor phenotypes did not differ significantly between adolescent- and adult-onset FMDs, with the exception of parkinsonism, which was observed only in the latter. Compared with adult-onset FMDs, adolescent-onset FMDs were associated with a longer disease duration, a higher number of medical consultations before diagnosis, and a higher frequency of functional seizures and infections, but with lower rates of insomnia, fatigue, and antipsychotic use. In multivariable analysis, adolescent-onset FMDs remained independently associated with a greater number of medical consultations (adjusted OR 1.07; 95% CI 1.02–1.13), the presence of functional seizures (adjusted OR 2.06; 95% CI 1.09–3.8), and with lower occurrence of insomnia (adjusted OR 0.49; 95% CI 0.27–0.92) and fatigue (adjusted OR 0.51; 95% CI 0.30–0.86). Pain was more likely to be associated with the combined FMDs phenotype.

**Conclusions:**

Adolescent-onset FMDs are common and are associated with several non-motor symptoms in tertiary movement disorders centers**.** Early and accurate diagnosis may help to reduce unnecessary investigations and inappropriate treatments.

**Supplementary Information:**

The online version contains supplementary material available at 10.1007/s00415-026-13761-w.

## Introduction

Functional Motor Disorders (FMDs) represent a significant diagnostic and therapeutic challenge in pediatric neurology, particularly among adolescents. These disorders are characterized by the presence of motor symptoms (e.g. weakness, tremor, dystonia, gait disturbances) and may be accompanied by other functional neurological disorders (FND) such as sensory impairments (e.g. vision, auditory dysfunction) and functional seizures [1]. Their clinical presentation is common but often similar to other neurological or psychiatric diseases, therefore, leading to frequent misdiagnoses [2] and diagnostic delays, which can extend up to 3.5 years in patients with functional seizures [3].

Estimates of pediatric FMDs prevalence vary widely (2.8%–23.1%). Although most studies report a female predominance [1, 4–7], others have described a higher proportion of boys, with rates up to 63.6% [1, 8–10], highlighting the marked heterogeneity of pediatric samples and diagnostic approaches. For example, different diagnostic criteria have been applied—including “conversion/psychogenic movement disorder” [4, 10–15] or 'probable,' or 'possible' FMDs [5–7, 10, 13, 15]- often without evaluation by a movement disorder neurologist or pediatric neurologist [1]. The studied populations included pediatric patients aged 3 to 21 years old [4–6, 9–12, 14–16], although the FMDs diagnosis at early developmental stages is particularly challenging due to the complex interplay of developmental, psychological, and social factors [17, 18]. In addition, data have been collected in different clinical settings such as pediatric emergency departments [14, 16], general pediatric hospitals [5, 10, 11] and/or movement disorders clinics [4, 6, 13]. and the design of the study was often retrospective [4, 6, 7, 11–13] frequently involving small sample sizes. [4–6, 10, 12, 13, 15]. Finally, symptoms such as pain and fatigue, though often reported by patients, were not systematically documented.

Therefore, the characterization of FMDs/FNDs in the pediatric population remains incomplete, especially when patients are evaluated outside specialized movement disorder centers. Marked heterogeneity in study designs, diagnostic criteria, and clinical settings—together with evolving and multifaceted presentation of symptoms during adolescence—continues to complicate the distinction between FMDs and other neurological or neuropsychiatric conditions [2, 19–21]. This diagnostic uncertainty likely contributes to delays in recognition and treatment, with potentially negative consequences for clinical outcomes and long-term disability [2].

However, no large multicenter study using a definite FMDs diagnosis has systematically characterized adolescent-onset cases, compared adolescents with adult-onset patients, or examined the clinical correlates of isolated versus combined motor phenotypes in this age group. To address these gaps, the present study characterizes adolescent-onset FMDs using the WHO definition of adolescence and a definite diagnosis established by movement disorder specialists. We further examine the frequency of isolated and combined motor phenotypes and evaluate their associations with demographic and clinical variables, co-occurring FNDs, and neurological or psychiatric comorbidities.

## Methods

### Study design

In this observational cross-sectional study, data were extracted from the Italian Registry of Functional Motor Disorders (IRFMDs), managed by the Department of Neurosciences, Biomedicine and Movement Sciences, University of Verona, and by the Italian Academy for the Study of Parkinson’s Disease and Other Movement Disorders (Accademia LIMPE DISMOV RADAC project) and Fondazione LIMPE. Full methods of the IRFMDs are reported elsewhere [22].

### Subjects

Consecutive outpatients with FMDs were recruited from 25 tertiary movement disorders centers. Inclusion criteria were as follows: age at FMDs > = 10  years; a clinically definite diagnosis of FMD based on Gupta and Lang criteria, with the presence of positive signs and distractibility maneuvers [23]; and the presence of one or more FMDs phenotype including tremor, dystonia, weakness, gait disorders, jerks, facial motor disorders, and Parkinsonism. Exclusion criteria included any cognitive or physical impairment that precluded the ability to provide an informed consent.

At each participating center, a neurologist expert in movement disorders assessed patients in a single session, confirmed the diagnosis of FMDs, and conducted a structured interview including several demographical and clinical variables. [] [22]Demographic data included age and gender, while clinical variables comprised age at FMDs onset and disease duration, the number of medical consultations before the definite diagnosis of FMDs, FMDs phenotypes, such as weakness, dystonia, jerks, tremor, facial disorders and tics, and gait disorders. We recorded the presence of FMD phenotype as well as patients’ self-reported nonmotor symptoms, including pain, headache, insomnia (defined as patient-reported difficulty falling asleep, maintaining sleep, or experiencing non-restorative sleep, in line with commonly accepted clinical definitions) [24], panic attacks, depersonalization/derealization, anxiety, and fatigue). Additional information included the presence of other FNDs, such as functional seizures, sensory and visual functional symptoms, as reported during the structured clinical interview. The assessment also included assessment for psychiatric and neurological comorbidities; predisposing and precipitating factors (e.g. physical and psychological trauma); family history of neurological disease; the presence of friends affected by neurological diseases; and therapeutic interventions (e.g. medications, physiotherapy and other modalities of intervention).

We defined adolescence as the age range between 10 and 19 years, according to the World Health Organization (WHO) definition [25], and stratified the total sample into two groups: the adolescent-onset FMDs group (age at onset ≥ 10 and < 20 years) and the adult-onset FMDs group (age at onset ≥ 20 years). Age at FMDs onset was considered as the year of the first clinical manifestation of FMDs, as reported by the patients during the interview. Patients were stratified according to age at FMD onset, regardless of their age at the time of evaluation. To explore potential phenotype-related differences in clinical and demographic characteristics, we further stratified the adolescent-onset FMDs population into two groups: patients with a single FMD phenotype (isolated FMD) and those with multiple FMDs phenotypes (combined FMDs; e.g., dystonia plus tremor). Within the adolescent-onset FMDs subgroup, we also compared the distribution of motor phenotypes and the presence of functional seizures. The study was approved by the local ethics committee of the coordinator center (University of Verona, Azienda Ospedaliera Universitaria Integrata Verona, Prog. 1757CESC) and confirmed by the ethical committees of each participating center. All patients (or their guardians) were informed about the nature of the study and gave their written consent (consent for research). Patients were free to withdraw from the registry at any time.

### Statistical analysis

Data are expressed as mean ± standard deviation (SD) and range for continuous variables, counts, and percentages for categorical variables. For group comparisons, we employed an unpaired t-test for continuous variables and the chi-square test or Fisher’s test (in case of expected frequencies < 5) for categorical variables. In the adolescent-onset FMDs subgroup, we compared the various motor phenotypes and the presence of functional seizures using the chi-square test or Fisher’s test. Logistic regression models were created to estimate the unadjusted and adjusted odds ratio (OR 95% confidence interval [CI]) of adolescent-onset FMDs (dependent variable) in relation to sociodemographic and clinical characteristics (independent variables). In the adolescent-onset FMDs subgroup, we ran a logistic regression model to estimate the unadjusted and adjusted odds ratio (OR 95% confidence interval [CI]) for combined FMDs (dependent variable) in relation to sociodemographic and clinical characteristics (independent variables). Independent variables were chosen according to exploratory analysis results, clinical relevance, and sample size. For all comparisons, p-values less than 0.05 were statistically significant. Statistical analyses were performed using *SPSS* statistical software (version 25; IBM-SPSS, Armonk, NY, USA).

## Results

### Clinical characteristics of the adolescent-onset FMDs group

From a total of 847 patients with FMDs, 93 (10.9%) were included in the adolescent-onset FMDs group. Of these patients, 80.6% were female, with a mean age of 24.2 ± 11.3 years and a mean age at FMDs onset of 15.8 ± 2.6 years (Table 1). The most frequent phenotype was weakness (52.7%), followed by tremor (41.9%), dystonia (30.1%), and jerks (18.3%). In the adolescent-onset FMDs group, 50 patients (53.7%) presented with a combined phenomenology. As already noted, more than half of the patients in this group (*n* = 49, 52.7%) had weakness. Among these, weakness was isolated in 15/49 (30.6%) patients and combined with other phenotypes in 34/49 (69.4%). Overall, the distribution of weakness in adolescents-onset FMD was as follows: 44/49 (89.7%) had lower limb involvement, 19/49 (38.7%) had upper limb involvement, and 2/49 (4.1%) reported truncal weakness.

**Table 1:** Comparison of demographic and clinical features of adolescent-onset FMDs group and adult-onset FMDs group

	Adolescent-onset FMDs (*n*. 93)	Adult-onset FMDs (*n*. 754)	*P*-value
Female sex, *n* (%)	75 (80.6)	538 (71.4)	0.059
Age, *y*, mean (SD)	24. 2 ± 11.3	48.6 ± 14.5	** < 0.001**
Age at FMDs onset, y, mean (SD)	15.8 ± 2.6	44.4 ± 14.4	** < 0.001**
FMDs duration, *y*, mean (SD)	8.4 ± 11.3	4.2 ± 5.3	** < 0.001**
Medical consultations before the diagnosis, *n*, mean (SD)	4.4 ± 5.9	3.4 ± 3.4	**0.026**
FMD isolated phenotype, *n* (%)	43 (46.2)	344 (45.6)	0.91
*FMD phenotype*			
Tremor, *n* (%)	39 (41.9)	306 (40.6)	0.8
Weakness, *n* (%)	49 (52.7)	386 (51.2)	0.79
Dystonia, *n* (%)	28 (30.1)	186 (24.7)	0.25
Jerks, *n* (%)	17 (18.3)	86 (11.4)	0.056
Facial motor disorders, *n* (%)	13 (14.0)	99 (13.1)	0.82
Parkinsonism, *n* (%)	0	42 (5.6)	** < 0.001**
Gait disorders, *n* (%)	27 (29)	271 (35.9)	0.18
Acute FMDs onset phenotype, *n* (%)	68 (73.9)	544 (72.7)	0.81
FMDs spontaneous remission, *n* (%)	51 (58)	348 (48.5)	0.09
*Self-reported non-motor symptoms*			
Pain, *n* (%)	44 (47.3)	373 (49.5)	0.69
Migraine/headache, *n* (%)	31 (33.3)	258 (34.2)	0.86
Insomnia, *n* (%)	18 (19.4)	246 (32.6)	**0.009**
Panic attack, *n* (%)	18 (19.4)	126 (16.7)	0.52
Dissociation/depersonalization, *n* (%)	16 (17.2)	82 (10.9)	0.072
Anxiety, *n* (%)	39 (41.9)	383 (50.8)	0.107
Fatigue, *n* (%)	39 (41.9)	399 (52.9)	**0.046**
*Associated other FNDs*			
Functional seizures, *n* (%)	20 (21.5)	93 (12.3)	**0.014**
Visual symptoms, *n* (%)	11 (11.8)	111 (14.7)	0.45
Cognitive disorders, *n* (%)	13 (14)	122 (16.2)	0.58
Sensitive symptoms, *n* (%)	29 (31.2)	234 (31)	0.97
Fibromyalgia, *n* (%)	7 (7.5)	91 (12.1)	0.19
Irritable bowel syndrome, *n* (%)	2 (2.2)	32 (4.2)	0.33
*Psychiatric comorbidities*			
Schizophrenia, *n* (%)	0	9 (1.2)	0.29
Bipolar Disorders, *n* (%)	1 (1.1)	16 (2.1)	0.49
Major Depression, *n* (%)	10 (10.8)	117 (15.5)	0.22
Anxiety disorders, *n* (%)	16 (17.2)	183 (24.3)	0.12
Impulse control disorder/obsessive compulsive disorder, *n* (%)	3 (3.2)	16 (2.1)	0.49
Fugue state, *n* (%)	3 (3.2)	13 (1.7)	0.31
Somatoform disorder, *n* (%)	8 (8.6)	32 (4.2)	0.06
Eating disorders, *n* (%)	3 (3.2)	16 (2.1)	0.49
Sexual dysfunction, *n* (%)	1 (1.1)	8 (1.1)	0.99
Gender dysphoria, *n* (%)	0	1 (0.1)	0.73
Personality disorders, *n* (%)	5 (5.4)	17 (2.3)	0.074
*Predisposing factors*			
Childhood physical trauma, *n* (%)	10 (10.8)	53 (7)	0.19
Childhood psychological trauma, *n* (%)	10 (10.8)	62 (9.2)	0.62
*Neurological comorbidities*			
Multiple Sclerosis, *n* (%)	1 (1.1)	11 (1.5)	0.77
Parkinsonism, *n* (%)	0	18 (2.4)	
Hyperkinetic movement disorders, *n* (%)	1 (1.1)	17 (2.3)	0.46
Neuropathy, *n* (%)	1 (1.1)	27 (3.6)	0.2
Epilepsy, *n* (%)	3 (3.2)	15 (2.0)	0.43
Cerebrovascular disease, *n* (%)	1 (1.1)	44 (5.8)	0.053
Migraine, *n* (%)	10 (10.8)	71 (9.4)	0.68
*Familiarity*			
Familiarity for neurological diseases, *n* (%)	17 (18.3)	197 (26.1)	0.1
Friends with neurological diseases, *n* (%)	3 (3.2)	61 (8.1)	0.09
*Precipitating factors*			
Physical trauma, *n* (%)	10 (10.8)	123 (16.3)	0.16
Psychological trauma, *n* (%)	24 (25.8)	200 (26.5)	0.88
Surgery, *n* (%)	10 (10.8)	110 (14.6)	0.32
General anesthesia, *n* (%)	5 (5.4)	52 (6.9)	0.58
Adverse drug reaction, *n* (%)	4 (4.3)	48 (6.4)	0.43
Infection, *n* (%)	8 (8.6)	27 (3.6)	**0.022**
*Therapy*			
Physiotherapy, *n* (%)	29 (31.2)	256 (34)	0.59
Cognitive behavioural therapy, *n* (%)	15 (16.1)	94 (12.5)	0.32
Hypnosis, *n* (%)	1 (1.1)	11 (1.5)	0.76
Transcranial Magnetic Stimulation, *n* (%)	1 (1.1)	13 (1.7)	0.64
Botulinum toxin injections, *n* (%)	7 (7.5)	80 (10.6)	0.35
Other therapy, *n* (%)	6 (6.5)	48 (6.4)	0.97
*Drugs intake*			
Antipsychotic drugs, *n* (%)	2 (2.2)	59 (7.8)	**0.046**
Benzodiazepines, *n* (%)	27 (29)	177 (23.5)	0.23
Antidepressant drugs, *n* (%)	25 (26.9)	229 (30.4)	0.48
Antiepileptic drugs, *n* (%)	16 (17.2)	127 (16.8)	0.93
Other drugs, *n* (%)	21 (22.6)	123 (16.3)	0.13

Bold indicates significant values; significant associations at *P* < 0.05

FMDs, functional motor disorders; SD, standard deviation; FNDs, functional neurological disorders

### Demographic and clinical features of the adolescent-onset and adult-onset FMDs groups

**Fig. 1:**
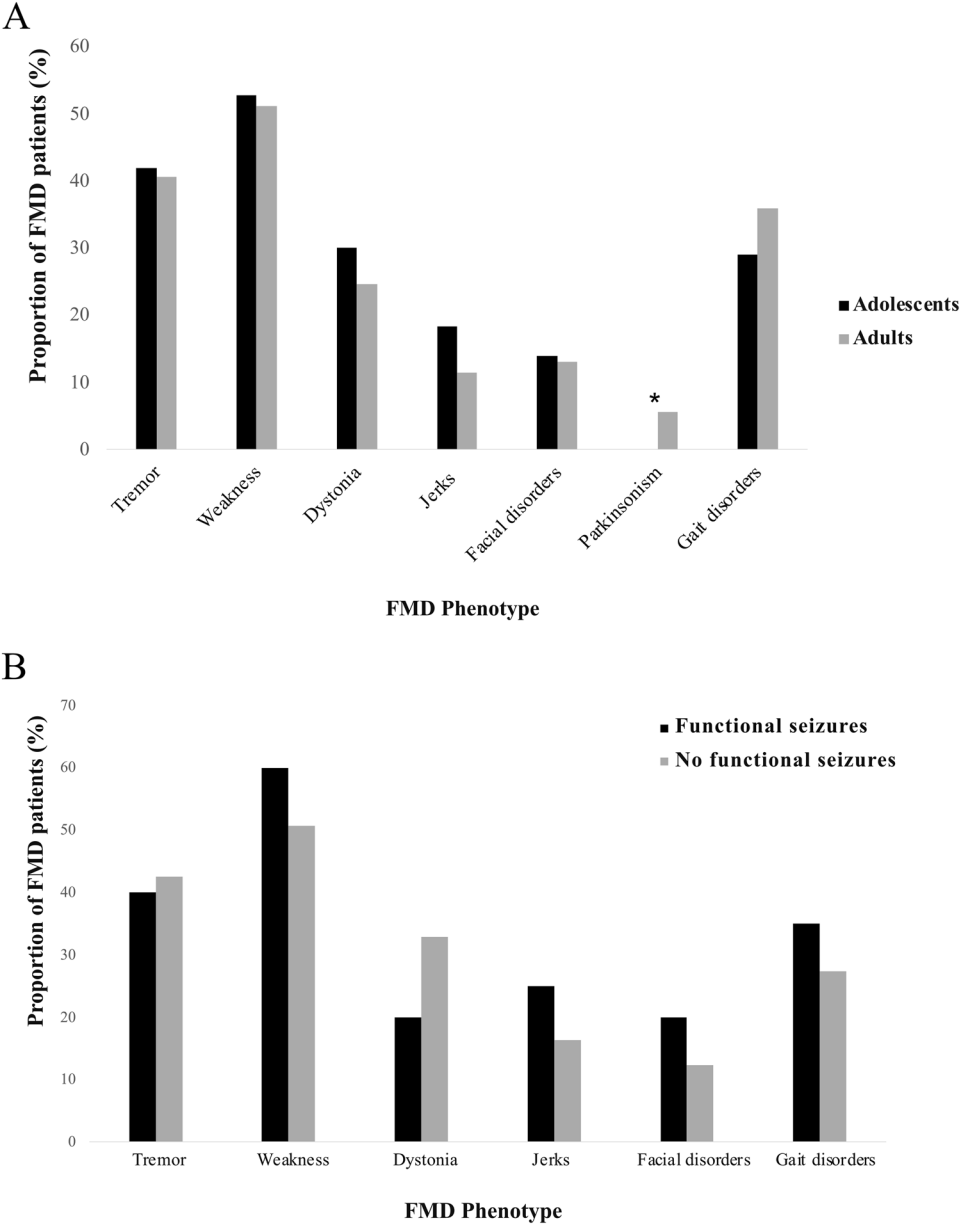
Panel A: percentage of FMDs in adolescent and adult-onset FMDs group; Panel B: percentage of FMDs in adolescent with and without functional seizures

When compared with the adult-onset FMDs group, the adolescent-onset FMDs group showed a longer FMDs duration (*p* < 0.001) and a higher number of medical consultations before receiving a diagnosis of FMDs (adolescents-onset FMD: range 1–30; adults: range 1–32) (*p* = 0.026), but a lower frequency of insomnia (*p* = 0.009), fatigue (*p* = 0.046), and antipsychotic drug use (*p* = 0.046). We did not find significant differences between the two groups in terms of FMDs phenotypes, with the exception of Parkinsonism (*p* < 0.001), which was more frequent in the adult-onset FMDs group (Table 1, Fig. 1A). The adolescent-onset FMDs group also showed a higher frequency of functional seizures (*p* = 0.014) as well as precipitating factors such as infections (*p* = 0.022) compared with the adult-onset FMDs group (Table 1). Among adolescent-onset FMD, no significant differences were found between motor phenotypes and the presence of functional seizures (Fig. 1B). After mutually adjusting for the variables reported in Table 1, the multivariate logistic regression model confirmed the association between adolescent-onset FMDs and the following variables: medical consultations before the definite diagnosis of FMDs (adjusted OR 1.07; 95% CI 1.02–1.13), presence of insomnia (adjusted OR 0.49; 95% CI 0.27–0.92), fatigue (adjusted OR 0.51; 95% CI 0.30–0.86), and functional seizures (adjusted OR 2.06; 95% CI 1.09–3.8) (Table 2).

**Table 2:** Clinical variables associated with adolescent-onset FMDs group

	Adjusted			
Independent variable	OR	95% CI		*P*-Value
Female vs. male sex^a^	1.41	0.769	2.61	0.26
Medical consultations before the diagnosis, n	1.07	1.02	1.13	**0.006**
Insomnia, yes vs. no^a^	0.49	0.27	0.92	**0.027**
Fatigue, yes vs. no^a^	0.51	0.3	0.86	**0.011**
Functional seizures, yes vs. no^a^	2.06	1.09	3.8	**0.024**
Infection, yes vs. no^a^	2.29	0.87	6.03	0.09
Antipsychotic drugs, yes vs. no^a^	0.26	0.06	1.14	0.07

Bold indicates significant values; significant associations at *P* < 0.05

FMDs, functional motor disorders; FNDs, functional neurological disorders; y, years

^a^Reference category; OR, odds ratio; CI, confidence interval

### Demographic and clinical features of isolated and combined adolescent-onset FMDs

In the exploratory analysis, when the adolescent-onset FMDs group was stratified into isolated and combined FMDs phenotypes, the combined phenotype (53.7%) was more common than the isolated one (46.3%). Adolescent-onset FMD with a combined FMDs phenotype had consulted a greater number of physicians before receiving a definite FMDs diagnosis (*p* = 0.01) and more frequently presented with tremor (*p* = 0.003), weakness (*p* = 0.001), dystonia (*p* = 0.002), jerks (*p* = 0.038), and gait disorders (*p* = 0.001). They also reported more frequent pain (*p* < 0.001), fatigue (*p* = 0.03), visual symptoms (*p* = 0.047), and childhood psychological trauma (*p* = 0.01). In addition, they more often underwent physiotherapy (*p* = 0.004) and cognitive behavioral therapy (*p* = 0.02) (Table 3).

**Table 3:** Comparison of demographic and clinical features of adolescent-onset FMDs group with isolated phenotype and those with combined phenotype

	Isolated (*n*. 43)	Combined (*n*. 50)	*P*-value
Female sex, *n* (%)	35 (81.4)	40 (80)	0.86
Age, *y*, mean (SD)	22.4 ± 9.8	25.8 ± 12.2	0.15
Age at FMD onset, *y*, mean (SD)	15.9 ± 2.8	15.8 ± 2.5	0.87
FMDs duration, *y*, mean (SD)	6.5 ± 9.8	9.9 ± 12.3	0.14
Medical consultations before the diagnosis, *n*, mean (SD)	2.6 ± 2.6	5.9 ± 7.4	**0.01**
*FMD phenotype*			
Tremor, *n* (%)	11 (25.6)	28 (56)	**0.003**
Weakness, *n* (%)	15 (34.9)	34 (68)	**0.001**
Dystonia, *n* (%)	6 (14)	22 (44)	**0.002**
Jerks, *n* (%)	4 (9.3)	13 (26)	**0.038**
Facial motor disorders, *n* (%)	4 (9.3)	9 (18)	0.22
Gait disorders, *n* (%)	1 (2.3)	26 (52)	** < 0.001**
Acute FMDs onset phenotype, *n* (%)	32 (76.2)	36 (72)	0.64
FMDs spontaneous remission, *n* (%)	27 (64.3)	24 (52.2)	0.25
*Self-reported non-motor symptoms*			
Pain, *n* (%)	12 (27.9)	32 (64)	** < 0.001**
Migraine/headache, *n* (%)	12 (27.9)	19 (38)	0.3
Insomnia, *n* (%)	5 (11.6)	13 (26)	0.08
Panic attack, *n* (%)	5 (11.6)	13 (26)	0.08
Dissociation/depersonalization, *n* (%)	8 (18.6)	8 (16)	0.74
Anxiety, *n* (%)	15 (34.9)	24 (48)	0.2
Fatigue, *n* (%)	13 (30.2)	26 (52)	**0.03**
*Associated other FNDs*			
Functional seizures, *n* (%)	11 (25.6)	9 (18)	0.37
Visual symptoms, *n* (%)	2 (4.7)	9 (18)	**0.047**
Cognitive disorders, *n* (%)	3 (7)	10 (20)	0.07
Sensitive symptoms, *n* (%)	10 (23.3)	19 (38)	0.12
Fibromyalgia, *n* (%)	1 (2.3)	6 (12)	0.07
Irritable bowel syndrome, *n* (%)	2 (4.7)	0	0.12
*Psychiatric comorbidities*			
Bipolar disorders, *n* (%)	0	1 (2)	0.35
Major Depression, *n* (%)	3 (7)	7 (14)	0.27
Anxiety disorders, *n* (%)	7 (16.3)	9 (18)	0.83
Impulse control disorder / obsessive compulsive disorder, *n* (%)	0	3 (6)	0.1
Fugue state, *n* (%)	2 (4.7)	1 (2)	0.47
Somatoform disorder, *n* (%)	2 (4.7)	6 (12)	0.2
Eating disorders, *n* (%)	1 (2.3)	2 (4)	0.64
Sexual disorders, *n* (%)	0	1 (2)	0.35
Personality disorders, *n* (%)	2 (4.7)	3 (6)	0.77
*Predisposing factors*			
Childhood physical trauma, *n* (%)	3 (7)	7 (14)	0.27
Childhood psychological trauma, *n* (%)	1 (2.3)	9 (18)	**0.01**
*Neurological comorbidities*			
Multiple Sclerosis, *n* (%)	0	1 (2)	0.35
Hyperkinetic movement disorders, *n* (%)	0	1 (2)	0.35
Neuropathy, *n* (%)	0	1 (2)	0.35
Epilepsy, *n* (%)	1 (2.3)	2 (4)	0.64
Cerebrovascular disease, *n* (%)	0	1 (2)	0.35
Migraine, *n* (%)	6 (14)	4 (8)	0.35
*Familiarity*			
Familiarity for neurological diseases, *n* (%)	11 (25.6)	6 (12)	0.09
Friends with neurological diseases, *n* (%)	1 (2.3)	2 (4)	0.65
*Precipitating factors*			
Physical trauma, *n* (%)	3 (7)	7 (14)	0.27
Psychological trauma, *n* (%)	12 (27.9)	12 (24)	0.66
Surgery, *n* (%)	5 (11.6)	5 (10)	0.8
General anesthesia, *n* (%)	1 (2.3)	4 (8)	0.22
Adverse drug reaction, *n* (%)	2 (4.7)	2 (4)	0.87
Infection, *n* (%)	6 (14)	2 (4)	0.88
*Therapy*			
Physiotherapy, *n* (%)	7 (16.3)	22 (44)	**0.004**
Cognitive Behavioural Therapy, *n* (%)	3 (7)	12 (24)	**0.02**
Hypnosis, *n* (%)	1 (2.3)	0	0.27
Transcranial Magnetic Stimulation, *n* (%)	0	1 (2)	0.35
Botulinum toxin injections, *n* (%)	1 (2.3)	6 (12)	0.07
Other therapy, *n* (%)	4 (9.3)	2 (4)	0.29
*Drugs intake*			
Antipsychotic drugs, *n* (%)	1 (2.3)	1 (2)	0.91
Benzodiazepines, *n* (%)	12 (27.9)	15 (30)	0.82
Antidepressant drugs, *n* (%)	12 (27.9)	13 (26)	0.83
Antiepileptic drugs, *n* (%)	8 (18.6)	8 (16)	0.74
Other drugs, *n* (%)	9 (20.9)	12 (24)	0.72

Bold indicates significant values; significant associations at *P* < 0.05

FMDs, functional motor disorders; SD, standard deviation; FNDs, functional neurological disorders

Variables associated with the combined FMDs phenotype at the univariable level were entered into a multivariable logistic regression model, excluding individual motor phenotypes to avoid definitional overlap. In the final model (Table 4), adolescents-onset FMD with a combined FMDs phenotype was independently associated with the presence of pain (adjusted OR 8.41; 95% CI 2.56–27.53) and with the number of medical consultations before the definite diagnosis of FMDs (adjusted OR 1.31; 95% CI 1.04–1.62).

**Table 4:** Clinical variables of adolescent-onset FMDs group associated with combined FMDs phenotype

	Adjusted			
Independent variable	OR	95% CI		*P*-value
Pain, yes vs. no^a^	8.41	2.56	27.53	** > 0.001**
Medical consultations before the diagnosis, *n*	1.31	1.04	1.62	**0.01**

Bold indicates significant values; significant associations at *P* < 0.05

FMDs, functional motor disorders; FNDs, functional neurological disorders; *y*, years

^a^Reference category; OR, odds ratio; CI, confidence interval

## Discussion

In this large multicenter study, we found that 10.9% of patients were adolescent-onset FMD who presented their first FMDs symptoms at a mean age of 15.8 years, a finding consistent with previous reports [1, 9]. Compared with our cohort, earlier studies estimated the prevalence of pediatric FMDs to range from 2.8% to 23.1%, with a higher proportion of cases observed in girls [1, 4–7, 11, 26]. Our findings provide an estimate of the proportion of adolescent-onset FMD within a large FMDs population accessing tertiary movement disorders centers for adults and confirm the higher frequency of females in this age group. This female predominance is similar to what has been reported in adult FMDs samples [7, 27, 28]. Within this context, we compared adolescent-onset FMD with patients whose FMDs onset occurred in adulthood. Multivariable regression analysis showed that adolescent-onset FMDs were associated with a higher number of medical consultations before receiving a definite diagnosis, a higher frequency of functional seizures, and a lower frequency of insomnia and fatigue. Our study highlights the relevance of recognizing FMDs during adolescence, a period in which diagnostic challenges may be amplified.

### Clinical characteristics of adolescent-onset FMDs

Functional weakness was the most frequently reported FMDs phenotype in both the adolescent-onset and adult-onset groups, which is consistent with previous studies [8, 26]. We did not find statistically significant differences between the two groups in any of the motor phenotypes, with the exception of Parkinsonism, which was observed only in the adult group. Parkinsonism, typically described in adults as a combination of bradykinesia, rest tremor, and rigidity, is rarely reported, or may be underreported, in adolescent-onset FMD. In contrast, some studies in pediatric populations have identified functional tremor (32.4%), dystonia (29.5%), and jerks (24.3%) as the most common clinical manifestations [1]. In line with these findings, tremor (41.9%) and dystonia (30.1%) were also frequently observed in our adolescent-onset FMDs cohort. Previous research suggests that tremor is more common in adults, whereas jerks may be more frequent in pediatric patients [9], but we did not observe significant differences between the two groups. A review estimated the prevalence of gait disturbances to be approximately 9.8% [1], although other studies have reported rates of up to 30%, while one case series described them in only 13% of patients [6]. In our cohort, gait disturbances were slightly more common, affecting about 29% of adolescent-onset FMD. Other findings have indicated that functional gait disturbances are significantly more prevalent in individuals with elderly-onset FMDs compared with younger patients [29].

Finally, because functional tic-like behaviors constitute a heterogeneous spectrum of abnormal movements whose clinical features overlap with primary tics (e.g., Tourette syndrome) [30, 31] and with other functional motor symptoms [32], their true prevalence remains uncertain. However, several studies have suggested a predominance in pediatric populations and in females [30, 33]. In our study, due to their low prevalence, these cases were grouped within the category of facial motor disorders, which represents a limitation of our work. Future studies should investigate functional tic-like behaviors using the detailed phenomenology only recently described in the literature [33]. Our patients typically experienced an acute or abrupt onset of FMDs with deterioration within a few days or weeks, consistent with previous reports. This pattern did not differ from that observed in the adult-onset FMDs group and has been associated with shorter symptom duration and a more favorable prognosis [1, 6, 9, 16, 34, 35]. In conclusion, we did not identify a phenotype specific to adolescent-onset FMD, suggesting that clinical features may not differ substantially across the lifespan.

Early diagnosis and the duration of FMDs are closely linked to patients’ prognosis. In our sample, the time from symptom onset to diagnosis was longer in the adolescent-onset group than in the adult-onset group. This may explain the significantly higher number of medical consultations before reaching a definitive FMDs diagnosis. Many of these patients may have initially been evaluated by pediatric neurologists who did not recognize FMDs, potentially contributing to a pattern in which patients continue to seek evaluation at different hospitals in search of a diagnosis or additional opinions. It is important to note that previous studies have reported a wide range in the time from symptom onset to diagnosis, varying from 10 days to 10 years (mean 20.5 months) [6, 13]. This variability reflects the complexity of diagnosing FMDs and highlights the need for prompt identification by pediatric neurologists with expertise in movement disorders, particularly in emergency settings or pediatric hospitals, where adolescents are more likely to be assessed. Early diagnosis is critical, as prognosis is considerably better in patients with a shorter symptom duration [13]. Psychiatric comorbidities, including anxiety, depression, obsessive–compulsive disorder, and neurodevelopmental conditions such as autism, are frequently observed in adolescents with FMD, reflecting the clinical complexity of this population [2, 4–8, 13, 20, 36]. Further complicating the clinical picture, several movement disorders can mimic FMDs, including paroxysmal kinesigenic dyskinesia [2], episodic ataxia, dopa-responsive dystonia, acute drug-induced dystonia, and Tourette syndrome, as well as various noninflammatory and inflammatory disorders [5, 10, 12]. Finally, in pediatrics, some diseases may initially present with psychiatric manifestations and only later develop movement disorders, as in Wilson disease [20].

Our data suggest that adolescent-onset-FMD were more likely to experience functional seizures than adult-onset FMD. These findings are in line with the results of the CODES trial (Cognitive Behavioural Therapy vs Standardised Medical Care for Adults With Dissociative Non‐Epileptic Seizures), which reported a predominantly young age at onset, with a modal age of 19 years and a median onset in the twenties [28, 37]. Previous studies using video-EEG in pediatric populations have reported a prevalence ranging from 3.5% to 20%, although it remains unclear whether this prevalence is underestimated compared with adults [38]. Functional seizures are paroxysmal events that may be mistaken for epileptic seizures, but they lack the EEG correlates typically observed in epilepsy [39]. Misdiagnosis can lead to diagnostic delays [40], unnecessary treatment with anti-seizure medications, additional investigations, and increased hospital visits [41, 42]. Functional seizures may also co-occur with epileptic seizures, with rates as high as 58% in patients with functional neurological disorders evaluated in tertiary epilepsy centers [38]. Previous studies focusing specifically on FNDs or mixed presentation have identified functional seizures as the most common dominant symptom, followed by weakness and sensory disturbances in children [43]. In our cohort, weakness was the most frequent symptom, and in adolescent-onset FMD functional seizures were not linked to specific motor phenotypes (Fig. 1B). Children with functional seizures have an increased risk of psychiatric disorders at the time of diagnosis and during the subsequent two years compared with children with epilepsy and healthy controls [44]. This finding highlights the importance of evaluating possible comorbid psychiatric conditions in this population [45–47]. Finally, once the diagnosis is made, a Mind–Body program involving a prompt biopsychosocial assessment and specific treatment should be implemented. With a prompt multidisciplinary intervention, most young people are able to return to full health and wellbeing. Early intervention is associated with better outcomes [48].

Among the non-motor symptoms, fatigue appeared to be more characteristic of the adult-onset FMDs group. In general, fatigue affects 45% to 93% of patients with FMDs [22, 49, 50], and in our sample it was more frequent in the adult-onset group (52.9%) and less common in the adolescent-onset group (41.9%). These findings are in line with a large international survey (*n* = 1048) reporting fatigue in up to 93% of patients with functional neurological disorders [51]. In our study, the lower occurrence of fatigue in adolescent-onset FMD may relate to the smaller sample size in this subgroup or to the challenges of identifying this symptom in adolescence, particularly when coexisting with psychiatric or neurological comorbidities. A significant proportion of FMDs patients experience fatigue, even when compared with individuals affected by other neurological disorders [50], and fatigue is strongly associated with reduced quality of life and lower self-rated health, regardless of FMDs severity. Its role should therefore be acknowledged in clinical practice and addressed with tailored interventions. The higher frequency of insomnia in the adult-onset FMDs group is consistent with previous reports, which indicate that sleep disturbances are common in adults, with rates up to 58% [52]. In pediatric populations, sleep disorders are not uncommon, but their prevalence has not been systematically investigated in a large cohort of adolescents with FMDs. To date, the literature does not clarify how fatigue and insomnia influence disability or quality of life in FMDs, nor which therapeutic strategies may be most effective in addressing them. Other non-motor comorbidities, such as migraine, have been reported in pediatric FND, suggesting a potential clinical overlap [53]. However, in our cohort, migraine frequency did not differ between adolescent-onset and adult-onset FMD.

### Clinical features and symptom burden in adolescent-onset FMD with combined FMDs

In the adolescent-onset FMDs group, we estimated the overall frequency of isolated and combined FMDs to examine their association with clinical variables. Some symptoms—weakness, tremor, and dystonia—occurred slightly more often in combination than in isolation. Several factors were associated with combined FMDs in adolescent-onset FMD, but only a few remained significant in the multivariate logistic regression analysis, namely the presence of pain and the number of medical consultations before diagnosis. These factors may reflect the diagnostic and therapeutic challenges encountered in patients with FMDs as well as the difficulties in assessing mixed or multifaceted phenotypes [54]. Previous studies have reported a wide range of prevalence rates for combined FMDs in pediatric populations, from 29 to 64%, although these studies differed in sample size and age ranges [13, 26].

The present study has both strengths and limitations. The main limitation of this study is that all participating centers were for adult at tertiary movement disorder clinics, which may have led to an underestimation of the prevalence of FMDs in the adolescent-onset FMD population. Within this context, our cohort may underrepresent milder or self-limiting cases that do not require specialist referral. Therefore, our findings may not be generalizable to the entire spectrum of adolescent-onset FMD. Another limitation is the cross-sectional design and the reliance on clinical records and patient self-reports, which may introduce recall bias. Moreover, some dichotomic variables (i.e. fatigue) may not adequately capture the complexity of symptoms, and the use of clinical scales would provide a more comprehensive assessment. However, a major strength of the study lies in the large, multicenter cohort, which is representative of the number of adolescent-onset FMD presenting to tertiary movement disorders centers across the Italian national territory. The standardized collection of clinical data across all centers allowed us to provide novel insights into FMDs in both adolescents-onset FMD and adult-onset FMD, using a definite diagnosis confirmed by neurologists specialized in movement disorders. Additional strengths include the use of 10 to 19 years as the cut-off for defining adolescent onset, in line with WHO recommendations [25], and the detailed characterization of psychiatric and neurological comorbidities.

In conclusion, these findings emphasize the importance of an accurate diagnosis in adolescent-onset FMD, as this can support the effective management of symptoms and the identification of associated psychiatric and neurological comorbidities. Collaboration between neurologists and pediatric neurologists specialized in movement disorders is encouraged to promote timely and appropriate diagnostic and therapeutic approaches. In adolescents, a missed or incorrect diagnosis may lead to significant consequences, including the initiation of unnecessary or potentially harmful treatments for alternative psychiatric or neurological conditions. These findings highlight the need for early diagnosis and prompt recognition of FMD in adolescents by pediatric neurologists, as well as pediatricians and general neurologists, who often represent the first point of clinical contact. Early and accurate diagnosis may help reduce unnecessary investigations and inappropriate treatments, while acknowledging that some functional symptoms in adolescents may follow a self-limiting course. Timely management may also help prevent progression toward more complex or combined FMDs presentations, but future longitudinal studies will be essential to determine whether early recognition and targeted interventions can modify the disease trajectory in adolescent-onset FMD.

## Supplementary Information

Below is the link to the electronic supplementary material.

Supplementary file1 (DOCX 21 KB)

## Data Availability

The datasets used and/or analysed during the current study are available from the corresponding author on reasonable request.
