# Supplementary material for: Clinical features of adolescent-onset functional motor disorders in tertiary movement disorders centers
Source: J Neurol. 2026 Mar 22;273(4):223. doi: 10.1007/s00415-026-13761-w (PMC13006462; doi:10.1007/s00415-026-13761-w)
Supplement: Supplementary file 1 — Supplementary file1 (DOCX 21 KB) [file 415_2026_13761_MOESM1_ESM.docx]

**Supplementary Appendix S1. Co-investigators of the Italian Registry of Functional Motor Disorders (IRFMDs) Study Group**

| **Name** | **Location** | **Role** | **Contribution** |
| --- | --- | --- | --- |
| Grazia Devigili | Fondazione IRCCS Istituto Neurologico Carlo Besta, Department of Clinical Neurosciences, Parkinson and Movement Disorders Unit, Milan, Italy. | Site Investigator | Site coordinator of data acquisition |
| Nico-Golfrè Andreasi | Fondazione IRCCS Istituto Neurologico Carlo Besta, Department of Clinical Neurosciences, Parkinson and Movement Disorders Unit, Milan, Italy. | Site Investigator | Site coordinator of data acquisition |
| Antonio Emanuele Elia | Fondazione IRCCS Istituto Neurologico Carlo Besta, Department of Clinical Neurosciences, Parkinson and Movement Disorders Unit, Milan, Italy. | Site Investigator | Site coordinator of data acquisition |
| Arianna Braccia | Fondazione IRCCS Istituto Neurologico Carlo Besta, Department of Clinical Neurosciences, Parkinson and Movement Disorders Unit, Milan, Italy. | Site Investigator | Site coordinator of data acquisition |
| Francesco Teatini, | FND outpatients clinic, Neurology and Stroke Unit, General Hospital of Bolzano, Bolzano, Italy. | Site Investigator | Site coordinator of data acquisition |
| Serena Salandin | Child Neurology and Neurorehabilitation Unit, Department of Pediatrics, Provincial Hospital of Bolzano (SABES-ASDAA), Lehrkrankenhaus der Paracelsus Medizinischen Privatuniversität, Bolzano-Bozen, Italy | Site Investigator | Site coordinator of data acquisition |
| Lucio Parmeggiani | Child Neurology and Neurorehabilitation Unit, Department of Pediatrics, Provincial Hospital of Bolzano (SABES-ASDAA), Lehrkrankenhaus der Paracelsus Medizinischen Privatuniversität, Bolzano-Bozen, Italy | Site Investigator | Site coordinator of data acquisition |
| Silvia La Spisa | Servizio di Neurologia e Riabilitazione dell’età evolutiva, Azienda Sanitaria dell’Alto Adige, Bolzano, Italia | Site Investigator | Site coordinator of data acquisition |
| Donatella Contrafatto | AOU Policlinico G. Rodolico - San Marco, Catania | Site Investigator | Site coordinator of data acquisition |
| Eleonora Chisari | AOU Policlinico G. Rodolico - San Marco, Catania | Site Investigator | Site coordinator of data acquisition |
| Lorena Belli | IRCCS Neuromed, Pozzilli, Italy. | Site Investigator | Site coordinator of data acquisition |
| Sara Pietracupa | IRCCS Neuromed, Pozzilli, Italy. | Site Investigator | Site coordinator of data acquisition |
| Immacolata Carotenuto | Center for Neurodegenerative Diseases (CEMAND), Department of Medicine, Surgery and Dentistry Scuola Medica Salernitana, University of Salerno, Baronissi (SA), Italy | Site Investigator | Site coordinator of data acquisition |
| Ferdinando Ambrosio | Department of Advanced Medical and Surgical Sciences, University of Campania “Luigi Vanvitelli”, Naples, Italy. | Site Investigator | Site coordinator of data acquisition |
| Oriana Ciaramaglia | Department of Advanced Medical and Surgical Sciences, University of Campania “Luigi Vanvitelli”, Naples, Italy. | Site Investigator | Site coordinator of data acquisition |
| Francesco Pagliuca | Department of Advanced Medical and Surgical Sciences, University of Campania “Luigi Vanvitelli”, Naples, Italy. | Site Investigator | Site coordinator of data acquisition |
| Sonia Mazzucchi | Centro Clinico Malattie NeuroDegenerative-Azienda Ospedaliero Universitaria Pisana. | Site Investigator | Site coordinator of data acquisition |
| Enrico Bergamin | Centro Clinico Malattie NeuroDegenerative-Azienda Ospedaliero Universitaria Pisana. | Site Investigator | Site coordinator of data acquisition |
| Ludovica Cori | Centro Clinico Malattie NeuroDegenerative-Azienda  Ospedaliero Universitaria Pisana. | Site Investigator | Site coordinator of data acquisition |
| Giovanna Savorgnan | S.C. Neurologia, Dipartimento di Area Medica Specialistica, ASST Pavia, Italia. | Site Investigator | Site coordinator of data acquisition |
| Mazza Sara | S.C. Neurologia, Dipartimento di Area Medica Specialistica, ASST Pavia, Italia. | Site Investigator | Site coordinator of data acquisition |
| Habetswallner Francesco | Clinical Neurophysiology Unit, Cardarelli Hospital, Naples, Italy. | Site Investigator | Site coordinator of data acquisition |
| Anny Votano | Botulinum Toxin Therapy Center, Neurology Unit, Academic Hospital, A.O.U. “R. Dulbecco”, Catanzaro, Italy; | Site Investigator | Site coordinator of data acquisition |
| Maria Paola Barillari | Botulinum Toxin Therapy Center, Neurology Unit, Academic Hospital, A.O.U. “R. Dulbecco”, Catanzaro, Italy; | Site Investigator | Site coordinator of data acquisition |
| Anna Rita Bentivoglio | UOC Neurologia, Fondazione Policlinico Universitario Agostino Gemelli IRCCS, Rome, Italy; Università Cattolica del Sacro Cuore, Rome, Italy. | Site Investigator | Site coordinator of data acquisition |
| Francesco Musso | Università Cattolica del Sacro Cuore, Rome, Italy. | Site Investigator | Site coordinator of data acquisition |
| Daniele Belvisi | Department Human Neurosciences, Sapienza, University of Rome, Italy and IRCCS Neuromed, Italy. | Site Investigator | Site coordinator of data acquisition |
| Matteo Costanzo | Department Human Neurosciences, Sapienza University of Rome, Italy and Department of Neuroscience, Istituto Superiore di Sanità, Rome, Italy | Site Investigator | Site coordinator of data acquisition |
| Matteo De Rosa | Clinica Neurologica Ospedale SS. Annunziata, Chieti. | Site Investigator | Site coordinator of data acquisition |
| Dario Calisi | Clinica Neurologica Ospedale SS. Annunziata, Chieti. | Site Investigator | Site coordinator of data acquisition |
| Tiziana De Santis | Department of Neurology, IRCCS Humanitas Research Hospital, Rozzano, Italy. | Site Investigator | Site coordinator of data acquisition |
| Antoniangela Cocco | Department of Neurology, IRCCS Humanitas Research Hospital, Rozzano, Italy. | Site Investigator | Site coordinator of data acquisition |
| Gabriele Imbalzano | Department of Neurosciences Rita Levi Montalcini, University of Turin, Turin, Italy.  SC Neurologia 2U, AOU Città della Salute e della Scienza, Torino, Italy. | Site Investigator | Site coordinator of data acquisition |
| Claudia Ledda | Department of Neurosciences Rita Levi Montalcini, University of Turin, Turin, Italy.  SC Neurologia 2U, AOU Città della Salute e della Scienza, Torino, Italy. | Site Investigator | Site coordinator of data acquisition |
| Paola Caruso | Department of Medical Surgical and Health Sciences Cattinara Hospital, University of Trieste, Trieste, Italy. | Site Investigator | Site coordinator of data acquisition |
| Luisa Sambati | IRCCS Istituto delle Scienze Neurologiche di Bologna, Bologna, Italy. | Site Investigator | Site coordinator of data acquisition |
| Francesca Valentino, | IRCCS Mondino Foundation, Pavia, Italy. | Site Investigator | Site coordinator of data acquisition |
| Piergiorgio Grillo | Department Brain and Behavioral Sciences, University of Pavia, Italy.  IRCCS Mondino Foundation, Pavia, Italy. | Site Investigator | Site coordinator of data acquisition |
